# 184 Use it or lose it: can engagement in physical activity close to one’s relative physical capacity protect against decline in physical functioning among older adults?

**DOI:** 10.1093/eurpub/ckae114.119

**Published:** 2024-09-26

**Authors:** Antti Löppönen, Katja Lindeman, Lotta Palmberg, Evelien Van Roie, Christophe Delecluse, Erja Portegijs, Taina Rantanen, Timo Rantalainen, Laura Karavirta

**Affiliations:** Faculty of Sport and Health Sciences and Gerontology Research Center, University of Jyväskylä, Jyväskylä, Finland; Faculty of Sport and Health Sciences and Gerontology Research Center, University of Jyväskylä, Jyväskylä, Finland; Faculty of Sport and Health Sciences and Gerontology Research Center, University of Jyväskylä, Jyväskylä, Finland; Department of Public Health, University of Turku and Turku University Hospital, Turku, Finland; Centre for Population Health Research, University of Turku and Turku University Hospital, Turku, Finland; Department of Movement Sciences, Physical Activity, Sports and Health Research Group, KU Leuven, Leuven, Belgium; Department of Movement Sciences, Physical Activity, Sports and Health Research Group, KU Leuven, Leuven, Belgium; University of Groningen, University Medical Center Groningen, Center of Human Movement Sciences, Groningen, the Netherlands; Faculty of Sport and Health Sciences and Gerontology Research Center, University of Jyväskylä, Jyväskylä, Finland; Faculty of Sport and Health Sciences and Gerontology Research Center, University of Jyväskylä, Jyväskylä, Finland; Faculty of Sport and Health Sciences and Gerontology Research Center, University of Jyväskylä, Jyväskylä, Finland

## Abstract

**Purpose:**

Physical activity (PA) is distinct from physical capacity (PC), even though they correlate strongly in old age. Physical capacity defines the boundaries for PA, while activities in daily life typically remain submaximal. Older people who approach their capacity in terms of intensity and duration of daily activities might be better protected from future declines in physical function compared to those who do not (i.e., “use it or lose it”), although prospective research is lacking to support this hypothesis. Therefore, this study compared changes in physical function over a four-year follow-up between community-dwelling older adults categorized based on their baseline PC and PA.

**Methods:**

This study is a longitudinal 4-year follow-up study in older adults aged 75-85 years at baseline (N = 311, 60% women). Baseline PC was measured by a 10-meter maximal walking speed, and PA was continuously monitored for 3-7 days using a thigh-worn accelerometer. PA intensity was examined using Mean Amplitude Deviation (MAD) and 5-second epochs. Baseline values of PA and PC were categorized distributionally (low & high) into lowPC-lowPA, lowPC-highPA, highPC-lowPA, and highPC-highPA profiles. Physical function was evaluated using the Short Physical Performance Battery (SPPB) at baseline and at the four-year follow-up, with total score and 5 x Sit-To-Stand (5xSTS) test time as the primary outcomes. Paired samples t-test and generalized estimating equations were used for analyses.

**Results:**

During the follow-up, the SPPB total score and the 5xSTS test decreased for all profiles (p<.05), except for the highPC-highPA profile (p=.055, p=.182, respectively). When comparing highPC-highPA to other profiles, a greater decrease in SPPB total score were observed in all other profiles (highPC-lowPA B -.62 (SE .22), p=.006; lowPC-highPA -.70 (.35), p=.045; lowPC-lowPA -1.86 (.28), p<.001) and in 5xSTS time for the lowPA profiles (highPC-lowPA 1.32 (.49), p=.004; lowPC-lowPA 1.92 (.67), p=.007).

**Conclusions:**

The findings suggest that engaging in PA close to one’s PC may help to protect against decline in physical functioning among older adults, especially among those with relatively high PC. Therefore, older adults should be encouraged to participate in physically challenging activities that could potentially improve their capacity.

